# Dual-Branch CNN for Direction-of-Arrival and Number-of-Sources Estimation

**DOI:** 10.3390/s26030809

**Published:** 2026-01-26

**Authors:** Yufeng Jiang, Lin Zou

**Affiliations:** 1 Glasgow College, University of Electronic Science and Technology of China, Chengdu 611731, China; 2022190901004@std.uestc.edu.cn; 2 School of Information and Communication Engineering, University of Electronic Science and Technology of China, Chengdu 611731, China

**Keywords:** direction of arrival (DOA), number of sources (NOS), Convolutional Neural Network (CNN), Multiple Signal Classification (MUSIC), noise subspace detection

## Abstract

Despite numerous conventional direction-of-arrival (DOA) methods, relationships between number of sources (NOS) and DOA are often ignored, which could yield meaningful estimation information. Therefore, a dual-branch Convolutional Neutral Network (CNN) integrated with squeeze-and-excitation (SE) blocks that can perform DOA and NOS estimation simultaneously is proposed to address such limitations. Extensive simulations demonstrate the superiority of the proposed model over several traditional algorithms, especially under low signal-to-noise (SNR) conditions, limited snapshots, and in closely spaced incident angle scenarios.

## 1. Introduction

Direction-of-arrival (DOA) estimation is a vital branch in signal processing and has drawn researchers’ attention worldwide, with applications in various fields, including wireless communications, sonar, radar, the Internet of Things (IoT), and electronic countermeasures [1,2,3]. To address such a topic, a myriad of solutions, including both traditional algorithms [4,5,6,7,8] and machine learning-based ones [9,10,11,12,13,14], have been proposed. The Minimum Variance Distortion Response (Capon) algorithm [5] can achieve high-resolution frequency wavenumber spectrum estimations while minimizing the power of interference and noise from other directions. The Multiple Signal Classification (MUSIC) algorithm [4] utilizes the orthogonality between the noise space and signal space. This algorithm is based on rotational invariance techniques (ESPRIT) [6]. The high-resolution approaches based on MUSIC are also actively applied in modern radar systems such as High-Frequency Surface Wave Radars (HFSWRs) [15,16] to improve the detectability of the target. Its variant Root-MUSIC algorithm [7] calculates roots of the polynomial from noise subspace, rather than meticulously searching for spectrum peaks, which reduces computational complexity. However, low signal-to-noise ratio (SNR) causes degradation in estimation performance, since formulaic- and array-structure-based algorithms such as MUSIC and ESPRIT are highly susceptible to disruption of signal and noise subspaces. Being one solution to such an issue, the Manifold Reconstruction Unitary ESPRIT (MR-UESPRIT) algorithm [17] presented by Veerendra et al. outperforms traditional methods in cases with varying SNR values, snapshots, and source number. In the current era of information explosion, conventional methods face the new problem of the number of incident signals approaching or even exceeding the number of array elements. G-MUSIC [8], another variant of MUSIC, is one solution that exploits random matrix theory (RMT) to ensure DOA estimation consistency even when the number of samples is as comparably large as that of array elements. Furthermore, most traditional DOA estimation algorithms require prior information about number of sources (NOS). An inaccurate number of signal sources would trigger a large error in DOA estimation given that the orthogonality between noise and signal subspaces would be severely affected. The Akaike information criterion-based method (AIC) [18] and minimum description length criterion-based method (MDL) [19] are offered as two of the most common NOS estimation approaches.

Despite the numerous classical DOA estimation methods, the perfect method has not yet been proposed. However, the current rapid development of Artificial Intelligence (AI) techniques offers a new orientation in DOA estimation: deep learning (DL). Deep learning-based methods generally demonstrate a preeminent estimation performance compared to that of traditional algorithms, especially when the quantity of data being used for training is sufficiently large and representative. A Multi-layer Artificial Neural Network (ANN) is utilized to automatically study the features contained, enabling end-to-end learning from raw input to complex output. Support Vector Machines (SVMs) [12,20] and Gaussian Mixture Models (GMMs) [21] are two neural networks with overfitting issues, which could be solved by several recently presented methods [11,12,13,14], based on Deep Neural Networks (DNNs) [9,10]. Among all types of ANNs, the Convolutional Neural Network (CNN) is the most prevalent. The CNN architecture is introduced to estimate DOA of multiple speakers [13] and a method based on CNN with Toeplitz Prior [14] achieves accurate direction finding when the source number exceeds the number of array elements. Nevertheless, a given NOS value is still needed as prior information.

Both conventional and DL-based methods either estimate DOA alone or treat NOS and DOA estimation as two separate processes, which fails to exploit critical correlations between these two estimation tasks. Therefore, a dual-branch CNN architecture is proposed in this paper, which addresses the limitation of conventional and DL-based methods by fully leveraging interrelations between DOA and NOS estimation tasks. It makes a fundamental shift by formulating two estimation tasks as a multi-task learning problem and is trained in an end-to-end manner. Moreover, squeeze-and-excitation (SE) blocks [22] and an RMT feature processor are novelly integrated into the network to improve the model’s robustness in a low-SNR scenario, where conventional methods perform poorly. A RMT feature vector and a covariance matrix of the received signals are taken as the input from which valuable shared features are extracted. To evaluate the effectiveness of the proposed architecture, comprehensive simulations on the dataset and in different scenarios of varying SNR, snapshots, and angular resolutions are conducted.

The paper is structured as follows: the signal model is described in Section 2; the proposed method is described in Section 3; the training process is detailed in Section 4; Section 5 presents simulation results; and Section 6 presents conclusions.

## 2. Signal Model

Among many types of array geometry, Uniform Linear Array (ULA) is the most fundamental and popular one, consisting of sensors equally distributed along a straight line. This geometry is the basis for several subspace-based algorithms, including MUSIC [4] and ESPRIT [6]. Meanwhile, L-shaped arrays or rectangular planner arrays are commonly applied in two-dimensional DOA estimation, where more complex geometries are required for resolving both azimuth and elevation angles. L-shaped arrays are usually composed of two orthogonal ULAs and rectangular planner arrays are arranged in a grid pattern. The most general array configuration is a non-uniform array, where the sensors’ locations are not restricted to a certain fixed pattern. Thus, it lacks a simple closed-form expression.

**Figure 1:**
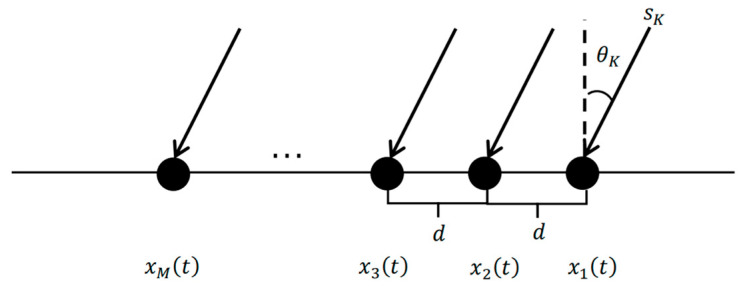
Structure of Uniform Linear Array (ULA). M is the number of the array elements; $x_{i}\left(t\right)$ is the received signal by $i^{th}$ array element at time t (i=1,2,…,M); d is the separation between the array element; $s_{K}$ donates far-field narrow-band signal with K sources; and $\theta_{K}$ represents the direction of arrival of $s_{K}$.

In this paper, an ULA is taken as an example for a fair and equitable comparison between the proposed method and conventional subspace-based algorithms like MUSIC and ESPRIT due to its generalizability. Its structure is illustrated in Figure 1.

Assume the number of the contained array elements is M and the separation between the array element is d. It is also assumed that K far-field narrow-band signals impinging on the array at time t are $s\left(t\right)=\left[s_{1},s_{2},\ldots ,s_{K}\right]$ from different directions represented by $\theta =\left[\theta_{1},\theta_{2},\ldots ,\theta_{K}\right]$. The received signals $x\left(t\right)$ are given by

(1) $$\begin{array}{c} \begin{array}{c} x\left(t\right)=A\left(\theta \right)s\left(t\right)+N\left(t\right),\;\;t=1,2,\ldots ,T, \end{array} \end{array}$$

where $N\left(t\right)$ represents additive Gaussian white noise, T is the number of snapshots, and $A\left(\theta \right)=\left[a\left(\theta_{1}\right),a\left(\theta_{2}\right),\ldots ,a\left(\theta_{K}\right)\right]$ is the array manifold matrix. $a\left(\theta_{K}\right)$ is the steering vector matrix of size M×1, being expressed as

(2) $$a\left(\theta_{K}\right)={\left[1,e^{j\frac{2\pi d}{\lambda }\sin\left(\theta_{K}\right)},e^{j\frac{2\pi d}{\lambda }\sin\left(\theta_{K}\right)2},\ldots ,e^{j\frac{2\pi d}{\lambda }\sin\left(\theta_{K}\right)\left(M-1\right)}\right]}^{T}.$$

Here, λ=c/f is the ratio between the light speed and the carrier frequency; j is the imaginary unit such that $j^{2}=-1$. The received signal’s covariance matrix $R_{xx}$ is calculated accordingly:

(3) $$R_{xx}=E\left[x\left(t\right){x\left(t\right)}^{H}\right]=A\left(\theta \right)R_{ss}A^{H}\left(\theta \right)+\sigma^{2}I_{M},$$

where $R_{ss}$ donates the covariance matrix of the source signals, $\sigma^{2}$ is the noise power, and $I_{M}$ is the identity matrix with size M. The expectation operator is E[·]; the conjugate transpose of the matrix is ${\left(\cdot \right)}^{H}$; and the transpose of the matrix is ${\left(\cdot \right)}^{T}$.

In conventional subspace-based algorithms, DOA and NOS estimation tasks are completed based on eigenvalue decomposition of $R_{xx}$ to separate signal and noise subspaces. By contrast, in the proposed method described in Section 3, a tensor ${\tilde{R}}_{xx}\in C^{2\times M\times M}$ serves as a direct input for the shared backbone, where the first channel is the real part of $R_{xx}$’s estimate and the second channel is its imaginary part. The proposed network can nonlinearly learn the encompassed features within the covariance matrix [23] for joint DOA and NOS estimation.

## 3. The Proposed Method

**Figure 2:**
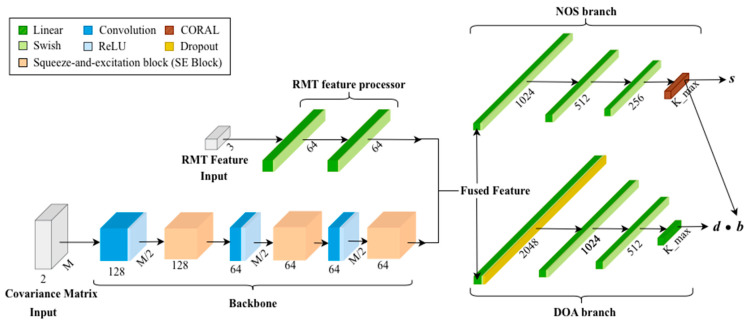
Overall architecture of the proposed dual-branch CNN for DOA and NOS estimation. Fused features derived from the shared CNN backbone and RMT feature processor are forwarded to NOS and DOA branches. NOS and DOA estimations are yielded, respectively, from these two branches. Different components are distinguished by color, as indicated by the legend in the top-left corner. Specifically, ‘Linear’ (green) indicates fully connected layer; ‘Swish’ (light green) indicates Swish activation function; ‘Convolution’ (blue) indicates convolutional layer; ‘ReLU’ (light blue) indicates ReLU activation function; ‘CORAL’ (brown) indicates Consistent Rank Logits (CORAL) [24] output layer; ‘Dropout’ (yellow) indicates dropout layer; and ‘Squeeze-and-excitation block’ (peach) indicate SE block [22]. Inputs are represented by gray blocks.

In this paper, a dual-branch Convolutional Neural Network (CNN) integrated with squeeze-and-excitation (SE) blocks [22] for simultaneous estimation of the number of sources (NOS) and direction of arrival (DOA) is proposed, which is depicted in Figure 2. The architecture can be divided into 4 parts: backbone, random matrix theory (RMT) feature processor, NOS, and DOA branches. The former two handle data preprocessed from raw signals to produce fused features. The latter two take the fused features as input to estimate NOS and DOA, respectively.

The covariance matrix is fed into the shared backbone (Section 3.2), passing three convolutional layers interleaved with three SE blocks to extract features. Moreover, a ReLU activation function is set after each convolutional layer. Meanwhile, RMT features (Section 3.1), being the other input, are fed into the RMT feature processor, passing two fully connected layers, followed by a Swish activation function for each. The outputs of the backbone and the RMT feature processor are then concatenated, forming ‘Fused Features’, which are further forwarded to the NOS and DOA branches for two estimation tasks. The NOS branch consists of three fully connected layers, Swish activation after each, followed by a CORAL output layer, whose principle is illustrated in detail in Section 3.3. The DOA branch encompasses four fully connected layers, with Swish activation after each. A dropout layer follows the Swish activation of the first fully connected layer, specifically. Its detailed principle is illustrated in Section 3.4.

### 3.1. RMT Feature Processor

Assume eigenvalues of covariance matrix $R_{xx}\in C^{M\times M}$ are $\lambda_{i}=\left[\lambda_{1},\lambda_{2},\ldots ,\lambda_{M}\right],\left(\lambda_{1}\geq \lambda_{2}\geq \ldots \geq \lambda_{M},i=1,2,\ldots ,M\right)$, the larger K eigenvalues represent signal space, and the smaller $\left(M-K\right)$ eigenvalues represent noise space. Input for RMT feature processor: RMT feature vector $f_{rmf}\in R^{3}$ is given by

(4) $$f_{rmt}=\left[\begin{array}{c} f_{1}\\ f_{2}\\ f_{3} \end{array}\right]=\left[\begin{array}{c} \frac{\lambda_{1}}{\lambda_{min}}\\ \frac{1}{K}\sum_{i=1}^{K}\lambda_{i}\\ \frac{1}{M-K}\sum_{i=K+1}^{M}\lambda_{i} \end{array}\right],$$

where $f_{1}$ is the conditional number, $f_{2}$ is the mean value of signal space eigenvalues, and $f_{3}$ is the mean value of noise space eigenvalues. Such features are processed by two fully connected layers, each with a Swish activation function, outputting a 64-dimensional representation.

### 3.2. Backbone

A tensor ${\tilde{R}}_{xx}\in C^{2\times M\times M}$ is the backbone input, which is derived from the real covariance matrix estimate ${\hat{R}}_{xx}$. The first channel of ${\tilde{R}}_{xx}$ is the real part of ${\hat{R}}_{xx}$, while the second channel is its imaginary part. The backbone consists of multiple convolutional layers, each being followed by batch normalization, ReLU activation, and an SE block [22].

Assume an output of a convolutional layer is a H×W×C tensor $U=[u_{1},u_{2},\ldots ,u_{c}]$. Within an SE block, the squeeze component performs global information aggregation utilizing global average filter:

(5) $$z_{c}=\frac{1}{H\times W}\sum_{i=1}^{H}\sum_{j=1}^{W}u_{c}\left(i,j\right).$$

Later, $z_{c}$ is initially compressed to C/r channels and then expanded to C channels through the excitation component, which contains two fully connected layers and an ReLU. r is the reduction rate. The final output of the SE block is obtained by multiplying the excitation segment output by U. Table 1 shows the detailed operation of each step in the backbone. The channel numbers are set as the power of two, balancing the model’s representational capability with its computational complexity. Table 2 shows the detailed structure of the SE block.

### 3.3. NOS Estimation Branch

Three fully connected layers with 1024, 512, and 256 channels, respectively, each being followed by a SiLU activation, are compressed in this branch. An extra functional block implementing ordinal regression through Consistent Rank Logits (CORAL) [24] is connected at the end. Features yielded from the three former fully connected layers are initially mapped to a constant c, based on which vector $s=\{s_{1},s_{2},s_{i},\ldots s_{K_{max}}\}$ is obtained by adding c with $K_{max}-1$ biases. The NOS estimation is given by [24]

(6) $$K=1+\sum_{i=1}^{K_{max}-1}{I\{b}_{i}>0.5\},$$

where $b=\sigma \left(s\right)=\{b_{1},b_{2},b_{i},\ldots ,b_{K_{max}}\}$ such that $b_{i}\in \{0,1\}$, and $\sigma \left(z\right)=1/(1+\exp\left(-z\right))$ is the Sigmoid function. $y=I\left\{\cdot \right\}$ means that y equals 1 if conditions inside the curly braces are true, otherwise 0.

### 3.4. DOA Estimation Branch

The DOA branch consists of 4 fully connected layers, each being followed by a SiLU activation. Moreover, a dropout layer (dropout rate of 0.4) is connected to the first dense layer. The number of channels of the former three fully connected layers is 2048, 1024, and 512, respectively. The last one yields a vector containing $K_{max}$ real values, which is the direction of the arrival signals. Let d donate this vector, such that $d=\{d_{1},d_{2},d_{i},\ldots ,d_{K_{max}}\}$. The final estimated DOA is $d_{i}=d_{i}\cdot I\{b_{i}>0.5\}$.

## 4. Training Approach and Settings

The generated dataset contains signals with a NOS (K) of $\left\{1,2,\ldots ,K_{max}\right\}$ under varying SNR conditions. Incident angles vary in the range of [−60°, +59°] and SNR values vary in the range of [−20, +20] dB (stride equals 1 dB). The maximum number of incident signals $K_{max}$ is set to 3. For single-source signals, samples are generated combining all possible incident angles with SNR values; double-source signals cover all possible 2-angle combinations from 120 angles, with each combination randomly selecting one SNR value; triple-source signals include all possible 3-angle combinations with random SNR selection. Samples are produced according to Equation (1) and a dataset including 292,900 samples is yielded, which is randomly split into 70% for training, 20% for validation, and 10% for testing. Considering that a training process utilizing such a dataset may be time-consuming, it is cut to contain only 133,745 samples. The number of snapshots of each sample is T=2000. Additionally, 10 array elements (M=10) are involved in this ULA, each having a distance d=0.15 from each other and wavelength λ=0.3. Samples are preprocessed into RMT features and covariance tensors as specified in Equations (3) and (4), respectively, for later training.

An Adam optimizer with a best default learning rate of 0.001 is adopted [25]. Nevertheless, after a fine-tunning process, the initial learning rate equaling 0.0005 provided the most optimal balance between coverage speed and model stability. Therefore, the model is trained with an initial learning rate equaling 0.0005, which declines by 0.7 every 10 epochs with 30 epochs in total for a 32-sample batch size. The model is optimized via applying the loss function below:

(7) $$L=\lambda_{1}\cdot L_{NOS}+\lambda_{2}\cdot L_{DOA},$$

where $\lambda_{1}=0.8$ and $\lambda_{2}=1.0$ are the weights for NOS loss function $L_{NOS}$ and DOA loss function $L_{DOA}$. These two loss functions are given by

(8) $$L_{NOS}\left(\hat{k},k\right)=-\frac{1}{N}\sum_{i=1}^{N}k_{i}\log\left({\sigma (\hat{k}}_{i})\right)+(1-k_{i})log(1-{\sigma (\hat{k}}_{i})),$$

(9) $$L_{DOA}\left(\hat{\theta },\theta \right)=\frac{1}{NK}\sum_{i=1}^{N}\sum_{k=1}^{K}{\left(\theta_{i}^{k}-{\hat{\theta }}_{i}^{k}\right)}^{2},$$

where $\hat{k},k,\hat{\theta },\theta$ are the estimated and ground-truth source numbers and the estimated and ground-truth incident angles. N is the batch size and K is the estimated source number from the NOS estimation branch. $\sigma \left(\cdot \right)$ is the Sigmoid function.

## 5. Simulation Results

Two measures, NOS accuracy and DOA root mean square error (RMSE), are used to numerically evaluate the proposed model’s estimation performance, as well as compare it with the performance of the classical algorithms (MUSIC [4], ESPRIT [6], Capon [5], Root-MUSIC [7]) under different conditions. DOA RMSE is given by Equation $\left(11\right)$ below and NOS accuracy is defined as the percentage of test samples where the estimated NOS value equals true NOS value, which is given by Equation (10).

(10) $$Accuracy_{NOS}=\frac{1}{N_{sample}}\sum_{i=1}^{N}I\left\{{\hat{k}}_{i}=k_{i}\right\},$$

where $N_{sample}$ is the total number of test samples; $k_{i}$ is the true number of sources; ${\hat{k}}_{i}$ is the estimated number of sources; and I{·} equals 1 if the inner condition is satisfied, otherwise 0.

(11) $$RMSE_{DOA}=\sqrt{\frac{1}{NK}\sum_{i=1}^{N}\sum_{k=1}^{K}{\left(\theta_{i}^{k}-{\hat{\theta }}_{i}^{k}\right)}^{2}},$$

where N is the batch size; K is the estimated source number; $\theta_{i}^{k}$ represents the true DOA value of the $i^{th}$ signal’s $k^{th}$ source in a batch; and ${\hat{\theta }}_{i}^{k}$ represents correspondant estimate of $\theta_{i}^{k}$.

### 5.1. Simulation on Test Dataset

**Figure 3:**
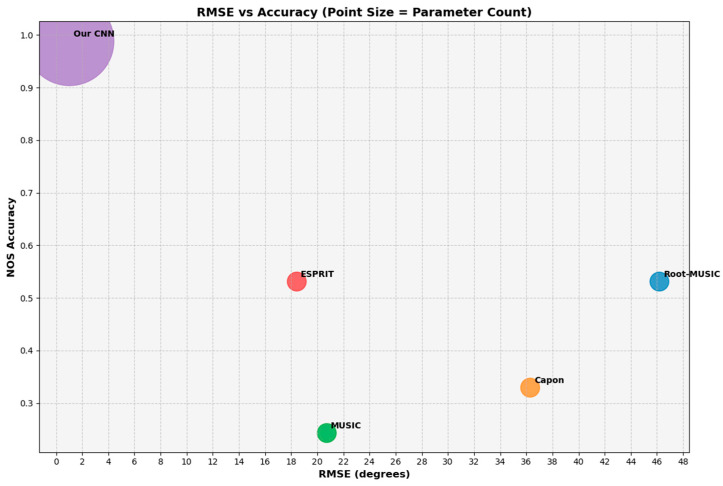
Ball chart of NOS accuracy versus DOA RMSE, reflecting estimation performance. The size of each ball is proportional to the number of model parameters.

The characteristics of the test dataset are similar to that of the training dataset, which contains signals with varying SNR values and source numbers less than $K_{max}$. The simulation results on the test dataset are depicted in Figure 3, which demonstrates the significant superiority of our CNN over other algorithms with higher NOS accuracy and lower DOA RMSE, despite its larger parameter number. Table 3 elucidates the simulation results specified by the source numbers when $K_{max}=3$.

Notably, an increasing trajectory can be observed in RMSE for most of the conventional algorithms as the number of sources rises, suggesting performance degradation and their increasingly limited estimation capability in more complex scenarios. By contrast, instead of performance degradation, our proposed data-driven approach demonstrates higher adaptability that achieves a lower DOA RMSE of 0.9376° when source number equals 3.

### 5.2. Simulation in Varying SNR Scenarios

In this scenario, samples with SNR values varying from −20 dB to 20 dB are contained, which is suitable for indicating a model’s generalization capability in practical NOS and DOA estimation tasks, where signals qualities are usually unknown. Low SNR values represent a harsh environment where noisy impacts are significant; high SNR values represent better-quality signals with less noisy disruption, through which the model’s noise immunity is evaluated. Incident angles and snapshots are set to {−20, 15, 40} degrees and 2000, respectively. Other parameters are the same as that in the test dataset. Given that classical algorithms only focus on DOA estimation, the minimum description length criterion-based method (MDL) [19] is adopted to estimate NOS.

**Figure 4:**
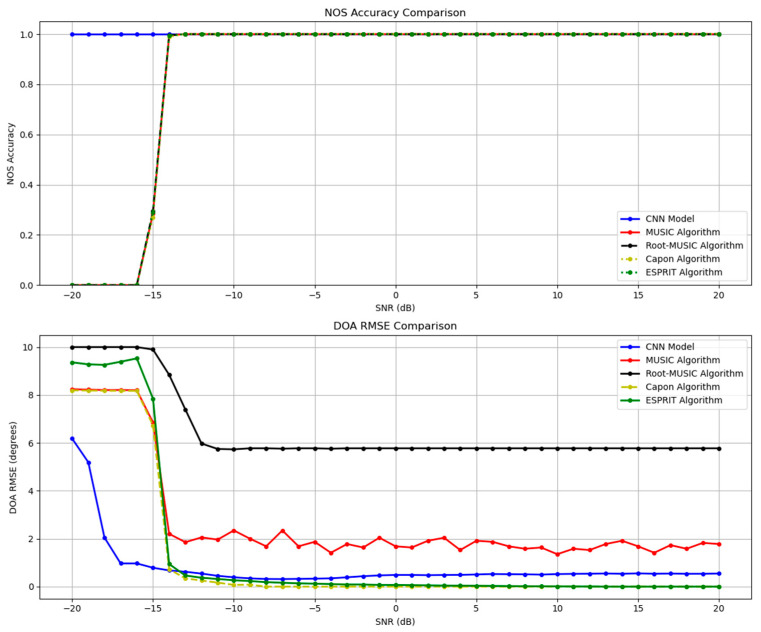
Line chart of NOS estimation accuracy and DOA estimation RMSE of applying different methods in scenarios with varying SNR values.

Performance declines generally for all methods in both estimation tasks as SNR value decreases, as illustrated clearly in Figure 4.

The proposed model achieves a NOS accuracy close to 1 and DOA RMSE approximately equaling 6 degrees when SNR = −20 dB, and remarkably outperforms conventional algorithms under extremely low SNRs between −20 and −15 dB. A sharp change in NOS accuracy at an SNR of about −15 dB for the conventional methods can be observed. This phenomenon is due to the intrinsic limitation of the standard MDL criterion ($MDL_{modified}\left(k\right)=-logL\left(k\right)+\alpha \cdot 0.5\cdot k\left(2M-k\right)logN$), where the penalty weight α is fixed to 1. This fixed value of α could lead to underestimation in NOS once SNR is below a certain threshold. In our simulation, such a threshold equals −15 dB, which is determined by several parameters such as the number of array elements (10, in this simulation) and snapshots (2000, in this simulation). Altering any of these parameters would cause a shift in threshold. Meanwhile, the proposed method stays at a consistent unity accuracy as it avoids the limitation of the standard MDL criterion by directly learning a nonlinear mapping from the input covariance matrix to both the NOS and DOAs. A sharp drop in DOA estimation at an SNR around −15 dB for all methods can also be observed. Conventional methods rely on accurate subspace separation when estimating DOA, where the first K largest eigenvalues represent signal subspace and the remaining (M−K) eigenvalues represent noise subspace. M is the number of array elements and K is the estimated number of sources from the standard MDL criterion. Therefore, the NOS estimation for conventional methods affects their DOA estimation. For conventional algorithms, this phenomenon is caused by the sharp change in NOS at SNR around −15 dB that has been discussed before. For the proposed CNN model, higher SNR provides clearer spatial features that are easier for the network to extract, so that its DOA RMSE decreases gradually as SNR increases from −20 to −15 dB. However, in a comparably higher-SNR environment, its DOA estimation ability is less preeminent, since deep networks will reach a floor RMSE value under the limitations of factors such as the scale of the training dataset [9].

### 5.3. Simulation in Varying Snapshots Scenarios

**Figure 5:**
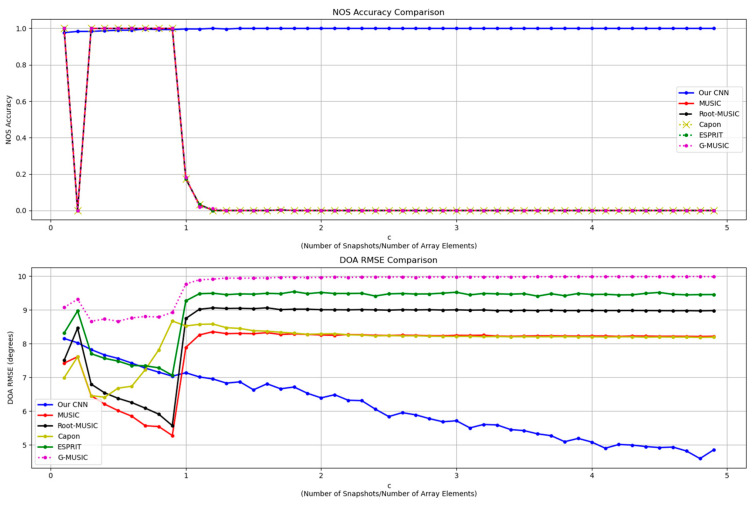
Line chart of NOS estimation accuracy and DOA estimation RMSE of applying different methods in scenarios with varying snapshots. ‘c’ represents the ratio between the number of snapshots and that of array elements.

This test set consists of signals with varying snapshots equaling c·M, where $c\in \left[0.1,\;5\right]$ with a stride of 0.1; M donates the number of array elements. SNR values are set to −10 dB for all signals and other characteristics of the incident angles are the same as that in the training dataset. The G-MUSIC [8] algorithm is additionally involved in this comparison, which is commonly applied in low-SNR and close-angle incident scenarios where the source number is not far lower than the array element number. A performance comparison is shown in Figure 5.

In terms of NOS estimation, the proposed model’s estimation accuracy is stably maintained at nearly 1. The first steep drop in accuracy of the subspace-based algorithms at c=0.2 is triggered by the singular-sample covariance matrix becoming rank-deficient and thus producing multiple zero eigenvalues. The second sudden drop is related to the value of a penalty weight α in the MDL criterion: $MDL_{modified}\left(k\right)=-logL\left(k\right)+\alpha \cdot 0.5\cdot k\left(2M-k\right)logN$. In the standard MDL criterion, α equals 1, which leads to underestimation under low-SNR condition. In such cases, a smaller penalty weight (e.g., 0.3) is recommended. Nevertheless, environmental conditions are usually unknown in practical applications. Therefore, the standard MDL criterion is exploited in model performance comparison. This reflects that the proposed model is less susceptible to limited snapshots under low-SNR conditions and therefore more applicable in harsh scenarios.

In terms of DOA estimation, as the number of snapshots continues to increase, the proposed model’s DOA RMSE consistently decreases, demonstrating more robust estimation performance compared to other traditional algorithms.

### 5.4. Simulation in Varying Angular Resolution Scenarios

This test set comprises samples with a fixed SNR value (−10 dB); the DOAs are within the interval: [0−n,0,0+n] degrees, where ‘n’ refers to the angular resolution or the angle difference between two adjacent angles with n∈[1,29] (stride: 1). Other characteristics are the same as that in the training dataset.

**Figure 6:**
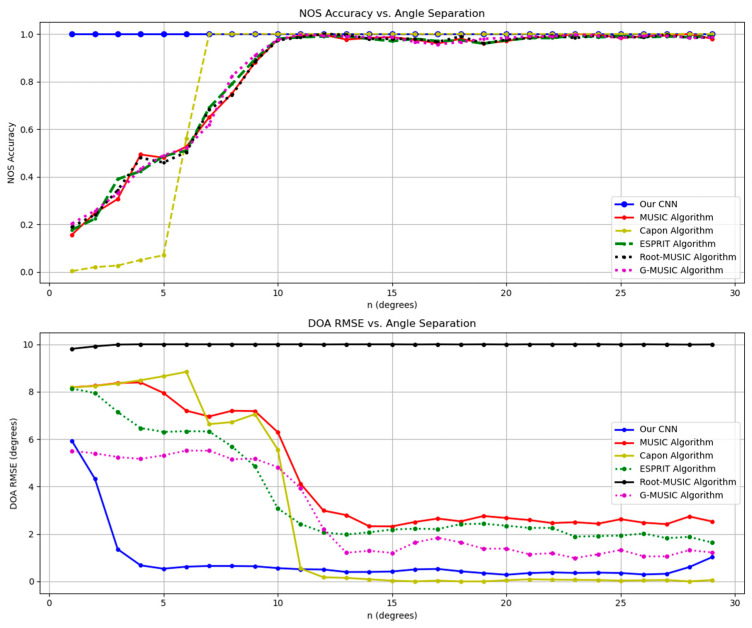
Line chart of NOS estimation accuracy and DOA estimation RMSE of applying different methods in scenarios with varying incident angular resolution.

As reported in Figure 6, the proposed method’s NOS estimation accuracy maintains stable at approximately 1, remarkably surpassing other algorithms, whose accuracies generally are below 0.2 at the point of n=1, and slowly reaches comparably high accuracy. Although the Capon algorithm’s accuracy converges to 1 faster among the conventional algorithms, a larger estimation error appears when n is less than or equal to 10.

For DOA estimation, the RMSE of our CNN declines quickly from about 6 degrees to below 2 degrees as n ranges from 1 to 5. Moreover, the RMSE of the proposed CNN remains at a similar value as n further increases. By contrast, the RMSEs of other algorithms are larger than that of the proposed model when the angle resolution is less than 11 degrees and converge thereafter. Though inaccuracy in DOA estimation using traditional algorithms can be attributed to an inaccurate estimated source number, to some extent, their DOA RMSEs are larger than the proposed CNN except for Capon even with a NOS accuracy approaching 1, which further substantiates the effectiveness and the superiority of our CNN in scenarios where noise is significant and the incident angles are narrowly distanced.

## 6. Conclusions

In this paper, a dual-branch CNN is proposed for joint estimation of NOS and DOA. By integrating a shared backbone and an RMT feature processor with two task-specific branches, the model effectively captures the inner relationships between source number and arrival angles. Experimental results across different scenarios confirm our model’s robustness under low SNR values, snapshots, and angular resolution conditions, as well as reveal its superiority over a set of classical algorithms.

For neural network-based DOA estimation method, exploring the lower bound of estimation bias is a meaningful research orientation. Therefore, future work should involve the investigation of the data-driven method-based DOA estimation method’s lower bound of estimation bias; constructing a theoretical framework for estimation accuracy that is suitable for such a method; and ultimately, advancing the field at a theoretical level. Furthermore, the signal models studied in this paper are based on narrow-band signals and AWGN channels. However, in real-world applications, wideband signal, colored noise, multi-path effects, significant power differences among sources, and mutual coupling between array elements are widely observed. These non-ideal phenomena lead to increased complexity in signal processing. To effectively address these issues, a new neural network model need to be developed—one that can not only adapt to the characteristics of wideband signals, but also, to the greatest extent, extract features from signal data under more complex environments where colored noise and multi-path effects persist. Thereby, received signals’ DOA can be accurately estimated. Thus, future research will also focus on developing more flexible and efficient neural network to resolve more complex DOA estimation problems.

## Figures and Tables

**Table 1:** Structure of backbone in the proposed dual-branch CNN. Each row specifies input size, operator, stride, and output size. Symbol ‘-’ means correspondent parameter is unnecessary. The channel numbers (e.g., 128, 64) are selected based on a common design paradigm.

Input Size	Operator	Stride	Output Size
2 × M × M	Conv2d	2	128 × M/2 × M/2
128 × M/2 × M/2	BatchNorm	-	128 × M/2 × M/2
128 × M/2 × M/2	ReLU	-	128 × M/2 × M/2
128 × M/2 × M/2	SE Block	-	128 × M/2 × M/2
128 × M/2 × M/2	Conv2d	1	64 × M/2 × M/2
64 × M/2 × M/2	BatchNorm	-	64 × M/2 × M/2
64 × M/2 × M/2	ReLU	-	64 × M/2 × M/2
64 × M/2 × M/2	SE Block	-	64 × M/2 × M/2
64 × M/2 × M/2	Conv2d	1	64 × M/2 × M/2
64 × M/2 × M/2	BatchNorm	-	64 × M/2 × M/2
64 × M/2 × M/2	ReLU	-	64 × M/2 × M/2
64 × M/2 × M/2	SE Block	-	64 × M/2 × M/2
64 × M/2 × M/2	Flatten	-	64 × M/2 × M/2

**Table 2:** Structure of SE block applied in the proposed backbone. *c* is the channel number being forwarded to the SE block and *r* is the reduction rate.

Input Size	Operator	Output Size
c × M/2 × M/2	AdaptiveAvgPool2d	c × M/2 × M/2
c × M/2 × M/2	Linear	c/r × M/2 × M/2
c/r × M/2 × M/2	ReLU	c/r × M/2 × M/2
c/r × M/2 × M/2	Linear	c × M/2 × M/2
c × M/2 × M/2	Sigmoid	c × M/2 × M/2

**Table 3:** DOA RMSE across various numbers of sources represented by K with the maximum source number being equal to 3. The unit is degree (°).

Methods	K = 1	K = 2	K = 3
Our CNN	5.9729	2.7994	0.9376
MUSIC [4]	3.0828	6.9456	13.5429
Capon [5]	24.9058	28.8082	25.9648
ESPRIT [6]	14.5110	18.5479	9.2892
Root-MUSIC [7]	59.5191	39.2725	35.2652

## Data Availability

Data are contained within the article.
